# A metabolically engineered bacterium controls autoimmunity and inflammation by remodeling the pro-inflammatory microenvironment

**DOI:** 10.1080/19490976.2022.2143222

**Published:** 2022-11-20

**Authors:** Jugal Kishore Das, Fengguang Guo, Carrie Hunt, Shelby Steinmeyer, Julia A Plocica, Koichi S. Kobayashi, Yufang Ding, Arul Jayaraman, Thomas A Ficht, Robert C. Alaniz, Paul de Figueiredo, Jianxun Song

**Affiliations:** a Department of Microbial Pathogenesis and Immunology, Texas A&M University Health Science Center, Bryan, TX, USA; b Department of Entomology, Texas A&M University, College Station, Bryan, TX, USA; c Department of Chemical Engineering, Texas A&M University, College Station, Bryan, TX, USA; d Department of Immunology, Graduate School of Medicine, Hokkaido University, Sapporo, Japan; e Department of Veterinary Pathobiology, Texas A&M University, College Station, Bryan, TX, USA

**Keywords:** Microbe, immunometabolism, regulatory T cells, autoimmunity, inflammation

## Abstract

Immunotherapy has led to impressive advances in the treatment of autoimmune and pro-inflammatory disorders; yet, its clinical outcomes remain limited by a variety of factors including the pro-inflammatory microenvironment (IME). Discovering effective immunomodulatory agents, and the mechanisms by which they control disease, will lead to innovative strategies for enhancing the effectiveness of current immunotherapeutic approaches. We have metabolically engineered an attenuated bacterial strain (i.e., *Brucella melitensis* 16M ∆*vjbR*, Bm∆*vjbR::tnaA*) to produce indole, a tryptophan metabolite that controls the fate and function of regulatory T (T_reg_) cells. We demonstrated that treatment with Bm∆*vjbR::tnaA* polarized macrophages (Mφ) which produced anti-inflammatory cytokines (e.g., IL-10) and promoted T_reg_ function; moreover, when combined with adoptive cell transfer (ACT) of T_reg_ cells, a single treatment with our engineered bacterial strain dramatically reduced the incidence and score of autoimmune arthritis and decreased joint damage. These findings show how a metabolically engineered bacterium can constitute a powerful vehicle for improving the efficacy of immunotherapy, defeating autoimmunity, and reducing inflammation by remodeling the IME and augmenting T_reg_ cell function.

## Introduction

Recent advances in cancer immunotherapy have not only revolutionized the field of tumor immunology,^1–3^ but also rejuvenated research into exploring novel strategies for autoimmune immunotherapy. Numerous studies have demonstrated promising results using immunotherapy to treat autoimmune and pro-inflammatory disorders.^4–6^ Nevertheless, despite advances in the field of autoimmune-immunotherapy, including regulatory T cell (T_reg_)-based therapy, the efficacy and benefits of these approaches remain less satisfactory due to limited *in vivo* T_reg_ expansion and persistence, the pro-inflammatory microenvironment (IME), and insufficient T_reg_ trafficking to inflamed sites. In addition, immune dysregulation of the IME contributes to disease.^7^ Immunotherapeutic combinations may produce greater efficacy, and thus strategies that circumvent these barriers are urgently needed.

To address this need, we developed and tested an intervention that combined two innovations. First, although a therapeutic role for live attenuated bacterial vaccines in addressing infectious diseases is undeniable and appreciated in cancer immunotherapy, the use of bacterial agents to manage autoimmune and pro-inflammatory diseases remains limited.^8–10^ Second, a growing number of microbiota-specific products and metabolites have novel immunologic properties that constitute one mechanism whereby the microbiota influence host health and disease.^11–14^ We therefore developed a metabolically engineered bacterial vaccine strain to produce immunomodulatory metabolites that improve autoimmunity and inflammation.

## Results

For the bacterial vector, we selected an attenuated strain of *Brucella melitensis* that harbors a deletion in *vjbR*, a master regulator of virulence (Bm∆*vjbR*).^15^ Like other Gram-negative organisms, *Brucella* strains express a lipopolysaccharide (LPS) lacking endotoxin activity. Importantly, Bm∆*vjbR* has been shown to be safe in immunocompetent and immunocompromised mice,^16^ goats,^17^ sheep,^18^ and non-human primates.^19^ We have also shown that Bm∆*vjbR* can combat cancer in a murine model by remodeling the tumor microenvironment (TME) to a pro-inflammatory state.^15^ Moreover, when Bm∆*vjbR* treatment was combined with adoptive cell transfer (ACT) of tumor antigen (Ag)-specific CD8^+^ T cells, tumor growth and proliferation were dramatically impaired.^15^ Conversely, in the current studies, we engineered Bm∆*vjbR* to express tryptophanase (tnaA); i.e., BmΔ*vjbR::tnaA*, to produce the tryptophan metabolite indole, a molecule that modulates the fate and function of T_regs_.^20^

**Figure 1.**
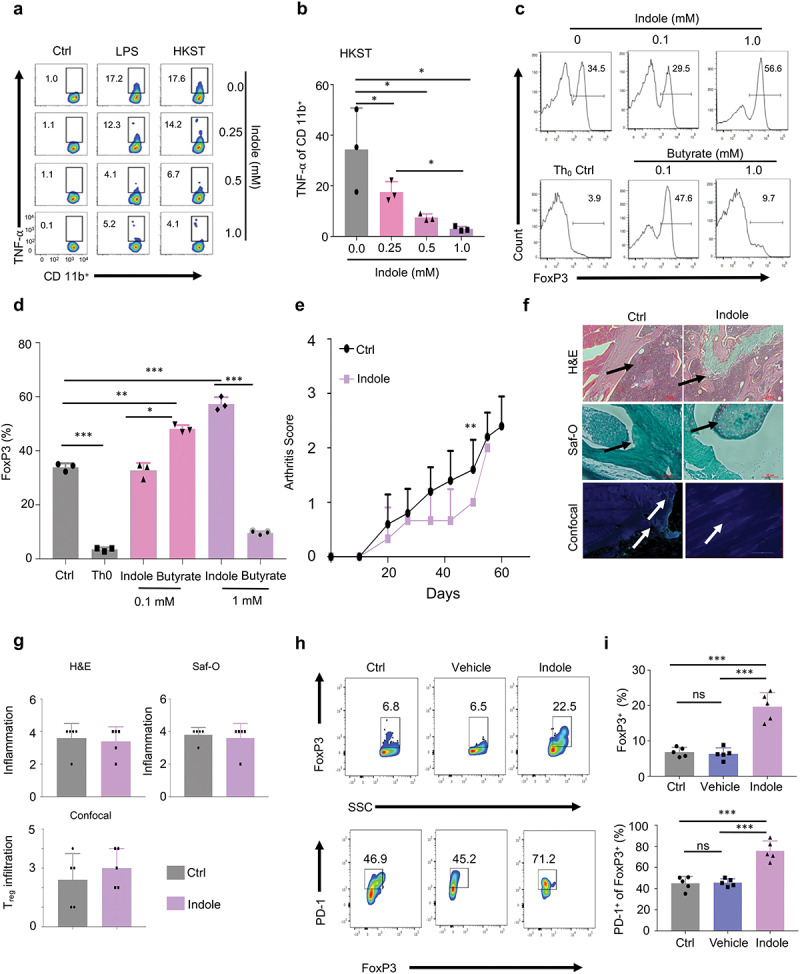
**Indole treatment dampens inflammation and promotes Treg cell expansion and activity**. a, Representative flow cytometric dot-plot analysis of the effect of indole on CD11b+ cells. 0.25, 0.5, or 1.0 mM indole was dissolved in DMF for the representative experimental flow cytometric analysis. b, Graphical representation of flow cytometric dot-plots derived from 3 independent experiments of heat killed Salmonella Typhimurium (HKST) group. c, flow cytometric histograms representing the dose-dependent indole induced differentiation of Treg cells. Experiment (N=3) was performed under Treg cell skew conditions ([TGF-β] = 2 ng/mL, [IL-2] = 100 U/mL). Th0 control represents non-Treg cell skew conditions. Butyrate was used as a control metabolite. d, Graphical representation of the effect of indole on the differentiation of Treg cells. e, Graphical representation of the effects of indole alone on CIA in mice (N=5). f, Representative images of H&E, Safranin O (Saf-O) stained tissues, and confocal microscopy of knee tissues of CIA mice on day 60 post induction of arthritis. Arrows indicate areas of infiltration of immune cells (H&E), cartilage-integrity (Saf-O) and Treg cell infiltration (confocal microscopy). Intracellular FoxP3 staining (green) was used to denote Treg cells. g, Quantitative analysis of H&E, Saf-O and Treg cell infiltration from confocal microscopy sections of Control (Ctrl) and indole-treated mice. h, Flow cytometric dot-plot analysis of PD-1 and FoxP3 in ex vivo activated CD4+ T cells isolated from LNs and spleen of C57BL/6 mice. Exposure to indole drives these cells towards higher Treg cell phenotype by increased FoxP3 expression. Vehicle (control) indicates corn-oil which was used as a solvent for indole. i, Graphical representation of FoxP3 derived from the flow cytometric dot-plots of CD4+ T cells exposed to indole. Graphical representation of PD-1+ FoxP3+ T cells (%) from the flow cytometric dot-plots. Data represent means ± SD. Student’s t-test or Tukey’s multiple comparisons test was applied for statistical analysis. *, **, ***: significance at p < 0.05, 0.01, 0.001.

We have reported that indole, when used at a range of physiologic concentrations, suppresses several inflammatory characteristics in immune and nonimmune cells,^21^ and also augments T_reg_ differentiation.^22^ Consistent with our previous reports, we demonstrated that indole suppressed TNF-αproduction in CD11b^+^ spleen cells after *E. coli* LPS (eLPS) and heat-inactivated *Salmonella* Typhimurium [HKST] stimulation (Figure 1a **&** Figure 1b) and dampened their activation by suppressing Akt and ERK signaling pathways in response to microbial agonists (eLPS and HKST) **(Fig. S1a)**. In addition, indole augmented the differentiation of naive CD4^+^CD25^−^ T cells into induced T_regs_ (iT_regs_) measured by FoxP3 *in vitro* in a dose dependent manner (Figure 1c **&** Figure 1d). These findings were consistent with our earlier reports,^15^ and were comparable to results from studies using the microbiota metabolite butyrate, albeit with distinct dose-dependency.^23^ Based on these findings, we hypothesized that indole would ameliorate immune-mediated inflammation in autoimmune and pro-inflammatory diseases.

**Figure 2.**
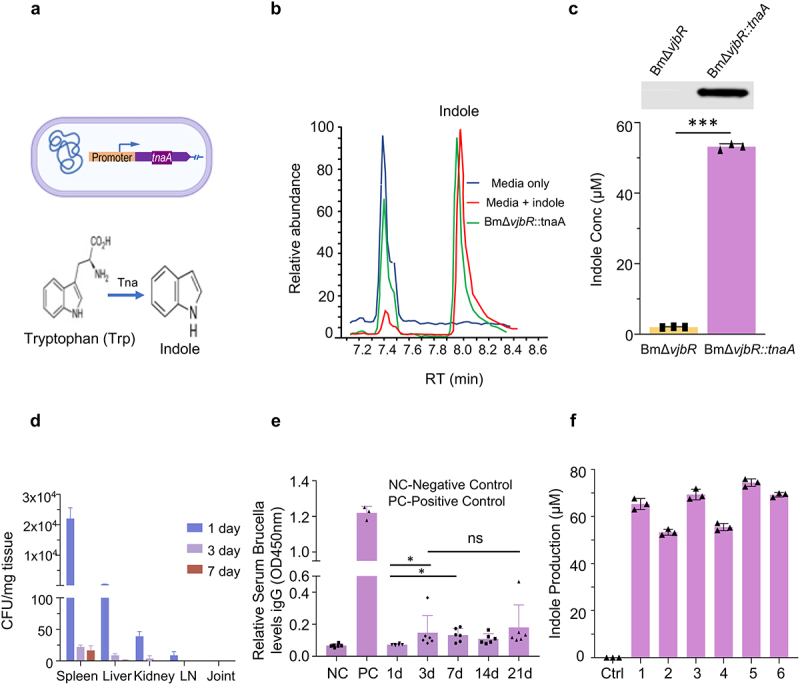
**BmΔ*vjbR* is engineered to produce indole**. a, Schematic representation of the engineered BmΔvjbR::tnaA harboring a plasmid carrying a tnaA expression cassette. The indole biosynthesis pathway is depicted in the figure. TnaA catalyzes the conversion of tryptophan to indole. b, Mass spectrometric analysis of indole production by engineered BmΔvjbR::tnaA. c, Western blotting analysis of the expression of tnaA protein in the parental strain compared with the engineered BmΔvjbR::tnaA strain. Graphical representation of the comparative analysis of indole production by BmΔvjBR parental bacterial strain and the engineered BmΔvjbR::tnaA strain. d, Colonization of engineered BmΔvjbR::tnaA in the spleen, liver, kidney and lymph-nodes of CIA mice. The bacteria colonized in all the organs for 3 days post-inoculation and could be observed only in the spleen for 7 days. The numbers represent single colonies grown on the plate. The bacteria recovered at 7 days post infection from mice still could produce indole. e, Serum ELISA analysis of anti-Brucella IgG production. The positive and negative controls were used as per the manufacturer’s instructions. f, Indole production of BmΔvjbR::tnaA bacteria recovered from CIA mice. Data represents means ± SD. Student’s t-test or Tukey’s multiple comparisons test was applied for statistical analysis. *, ***: significance at p < 0.05, 0.001.

**Figure 3.**
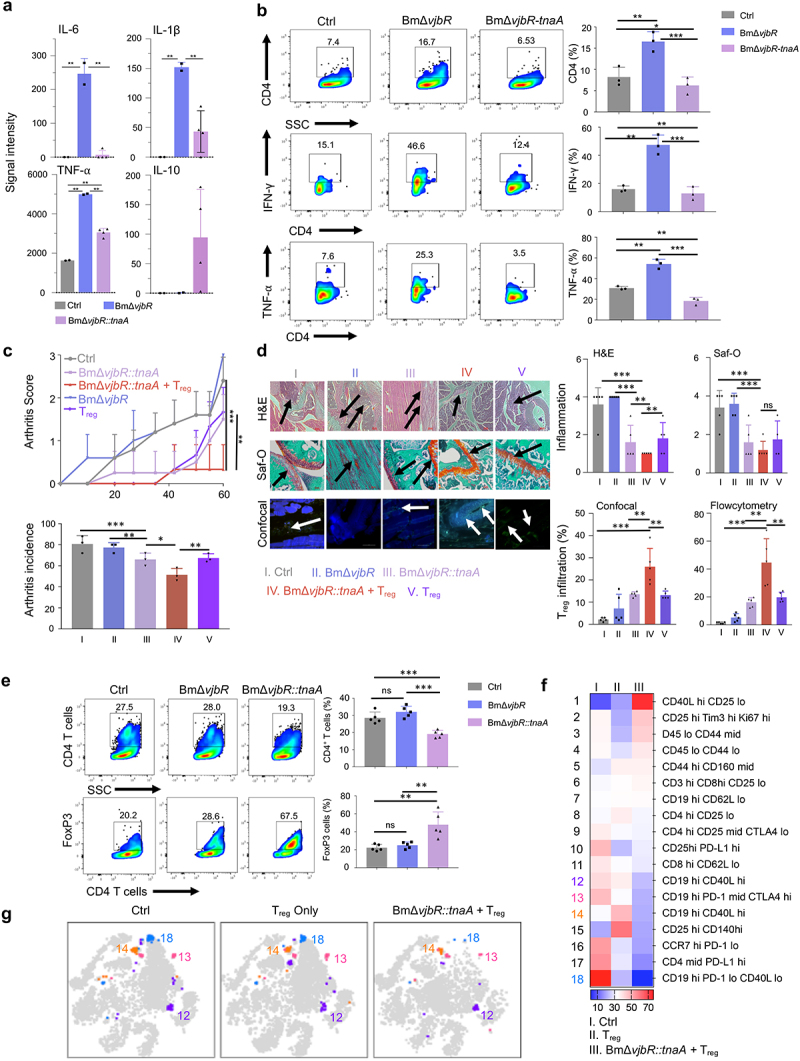
**BmΔ*vjbR::tnaA* significantly dampens inflammation and reduces arthritis in murine CIA model which is augmented by adoptive cell transfer (ACT) of Treg cells**. a, Cytokine arrays were used to measure pro-inflammatory cytokines produced by control, BmΔvjbR, and BmΔvjbR::tnaA treated BMDMs. b, Flow cytometric analysis of IFN-γ and TNF-α of T cells co-cultured with BMDMs. BMDMs were treated with either BmΔvjbR::tnaA or BmΔvjbR and then co-cultured with CD4+ T cells derived from pooled LNs and spleen of C57BL/6 mice for the assay. c, Arthritis score and arthritis incidence in CIA C57BL/6 mice from control (Ctrl, gray bar); BmΔvjbR (blue bar); BmΔvjbR::tnaA (pink bar); BmΔvjbR::tnaA followed by ACT of Treg cells (Treg, red bar) and ACT of Treg cells only; (N=5 in each group). d, Representative images of H&E, Saf-O staining, and confocal microscopy from mouse knees on day 60 post CIA induction. Arrows indicate areas of infiltration of immune cells (H&E), cartilage-integrity (Saf-O) and Treg cell infiltration (confocal microscopy). Intracellular FoxP3 staining (green) was used to denote Treg cells. Quantitative analysis of Treg cell infiltration and inflammation scores from these mice are also shown. e, Cells from knee and ankle joints were collected from CIA-induced mouse groups (Ctrl, BmΔvjbR, and BmΔvjbR::tnaA combined with ACT of Treg cells). These cells were then stained and quantified by flow cytometry using markers for CD4+ T cells (upper panel) and intracellular staining of FoxP3 (Treg cells) (lower panel). f, CIA-induced mice were treated with PBS (Ctrl), ACT of Treg cells only (Treg cells only; N=5), or BmΔvjbR::tnaA combined with ACT of Treg cells (N=5). Cells from the spleen, LNs and joints were stained with 21 markers and measured by CyTEK aurora flow cytometry. Heatmap shows immune cell profiles in different treatment groups of mice (scale bar represents percentage of cell in each treatment group within each cell type). g, viSNE map shows the four subtypes of B cells differentially expressed in the treated group of mice. Data represent means ± SD. Student’s t-test or Tukey's multiple comparisons test was applied for statistical analysis. *, **, ***: significance at p < 0.05, 0.01, 0.001.

Thus, we examined whether indole reduces autoimmune responses in a murine collagen-induced arthritis (CIA) model. We delivered 20 mg/kg indole in corn-oil to C57BL/6 mice (N = 5) having CIA and assessed the arthritis score in these mice compared to untreated control (PBS; N = 5). First, we showed that the severity of CIA was significantly attenuated in indole treated mice, which exhibited clinical scores of 0.8 ± 0.2 (means ± SEM) at (Day 50), compared to 1.6 ± 0.5 in controls (Figure 1e). However, a single dose of indole only showed a slight decrease in inflammation. Similarly, single dose treatment did not induce significant alterations in the infiltration of T_reg_ cells as assessed by confocal microscopy (Figure 1f **&** Figure 1g). These findings were in striking contrast to our *ex vivo* experimental findings, which indicated that indole significantly promoted the expansion of CD4^+^FoxP3^+^ T_reg_ cells and enhanced their activation by increased expression of PD-1 an immunosuppressive molecule, compared to the controls (*p* < .001), in cells derived from the mouse lymph nodes (LNs) and spleen (Figure 1h **&** Figure 1i). Based on our *in vivo* and *ex vivo* findings, we hypothesized that the sustained delivery of indole in a bacterial vector may greatly improve the durability of the molecule’s immunomodulatory effects, resulting in an attenuation of autoimmunity and inflammation in CIA. To test this hypothesis, we engineered a safe live-attenuated bacterial strain (i.e., BmΔ*vjbR::tnaA*) to constitutively produce indole (Figure 2a-c). First, we observed that the engineered bacterial strain survived mainly in the spleen for 7 days and liver and kidney for 3 dpi (Figure 2d). The bacteria did not penetrate the joints of the mice (Figure 2d). Second, we observed low-level immunogenicity by detection of anti-*Brucella* IgG antibodies from 3 to 21 dpi of the bacteria (Figure 2e). Moreover, we also determined that the colonies of BmΔ*vjbR::tnaA* bacterial strain recovered from mice challenged with collagen-induced arthritis (CIA) at 7 dpi could still produce indole (Figure 2f). Further, cytokine array profiling analyses showed that BmΔ*vjbR::tnaA* induced the expression of IL-10 in macrophages (Mφ) (Figure 3a **& Fig. S2a)**, which promotes the activities of T_reg_ cells and reduces autoimmunity and inflammation. Strikingly, BmΔ*vjbR::tnaA* also significantly (*p* < .01) reduced the expression of additional pro-inflammatory cytokines like IL-6, IL-1β and TNF-α in macrophages (Mφ) compared to BmΔ*vjbR* parental strain (Figure 3a). We also found that BmΔ*vjbR::tnaA*, when co-cultured with bone marrow-derived Mφ (BMDMs), not only significantly reduced the total CD4^+^ T cells (*p* < .001) but also reduced the production of the pro-inflammatory cytokines such as TNF-α and IFN-γ (*p* < .001) compared to the BmΔ*vjbR* parental strain (Figure 3b). Moreover, BmΔ*vjbR::tnaA* promoted the expansion of T_reg_ cells and significantly enhanced their activity as assessed by IL-10 production (*p* < .001) and PD-1 expression (*p* < .01) **(Fig. S2a**). Third, in the CIA model **(Fig S1b)**, a significant reduction in arthritis score and incidence was observed following treatment with BmΔ*vjbR::tnaA*. This amelioration of autoimmunity and inflammation was further augmented when BmΔ*vjbR::tnaA* treatment was combined with ACT of T_reg_ cells (Figure 3c). Fourth, we observed significantly (*p* < .01) reduced numbers of infiltrating inflammatory cells into the joints of mice treated with BmΔ*vjbR::tnaA*. This effect was further enhanced by BmΔ*vjbR::tnaA* treatment followed by the ACT of T_reg_ cells compared to the controls (*p* < .001) (Figure 3d). Finally, mice treated with BmΔ*vjbR::tnaA* showed reduced infiltrates in the joint and intact cartilages as evidenced by H&E analysis and Safranin O (Saf-O) staining of knee cross-sections (60 days post collagen administration). Notably, these findings were further attenuated by addition of ACT of T_reg_ cells (Figure 3d). There was also a significant reduction in the total CD4^+^ T cell proportion in the joints and a significant increase in T_reg_ cell proportion in mice treated with BmΔ*vjbR::tnaA* compared to controls (*p* < .001; Figure 3e). Since it is unlikely that BmΔ*vjbR::tnaA* bacteria will be used prior to the onset of arthritis in the clinic, we conducted an experiment to assess the impact of our strategy in treatment of established CIA by starting the administration of bacteria and T_reg_ cells, 3 weeks post collagen administration. Our results indicate that BmΔ*vjbR::tnaA* combined with ACT of T_reg_ cells significantly dampens the progression of the disease but is not sufficient to completely cure the disease **(Fig S3a and S3b)**.

**Figure 4.**
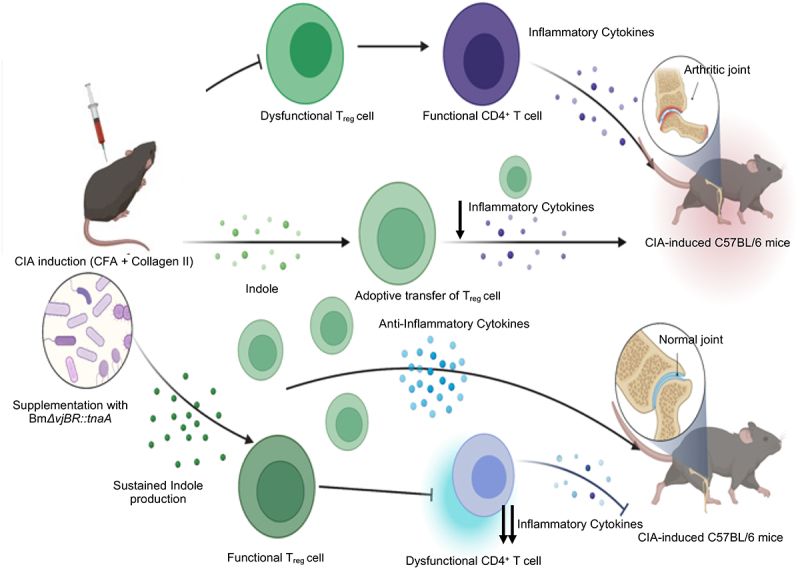
**Schematic representation of amelioration of CIA in C57BL/6 mice by engineered bacteria**. The illustration depicts the process of amelioration of CIA in C57BL/6 mice by the engineered BmΔvjbR::tnaA bacterial strain. C57BL/6 mice may harbor a microbiome deficient in production of the metabolite indole. When challenged with the CIA, these mice rapidly develop arthritis due to dysfunctional Tregs and rapid expansion of CD4+ effector T cells. However, when BmΔvjbR::tnaA bacteria are administered to mice bearing CIA, the Treg activity is substantially increased and the pathogenic activity of CD4+ effector T cells is compromised, which results in amelioration of joint inflammation and improved symptoms. This activity is greatly augmented by an ACT of T_regs_.

To identify the mechanism by which BmΔ*vjbR::tnaA* might be acting, we conducted a multiparametric CyTEK analysis from the cells isolated from the spleen, LNs and/or the knee and ankle joints of control, ACT with T_reg_ cells only, or ACT with T_reg_ cells plus BmΔ*vjbR::tnaA* groups. We found that the BmΔ*vjbR::tnaA* reduced the proportion of B cells (Figure 3f, Figure 3g**, Fig. S2b, and Table S1)** in addition to promoting T_reg_ cell expansion. Overall, our results indicate that BmΔ*vjbR::tnaA* remodels the IME and facilitates the expansion and suppressive function of T_reg_ cells as depicted in the illustration (Figure 4). Our results also indicate that BmΔ*vjbR::tnaA* may modulate B cell-mediated immunity in our murine model of CIA to alleviate symptoms of arthritis in these mice.

## Discussion

Several strategies have been employed to enhance the efficacy of live attenuated bacterial vaccines.^13,14^ However, bacterial vaccines producing immunomodulatory metabolites that alter the immunological tolerance and the IME have not been previously reported. Our work features both conceptual and methodological innovations. First, this work provides the first description of a live attenuated vaccine whose metabolism has been reprogrammed to amplify anti-autoimmune/inflammation activity.

Second, this study provides the first description of how combining a single dose of an engineered live attenuated bacterial vaccine strain with the ACT of T_reg_ cells can achieve potent therapeutic outcomes. Previous studies have demonstrated the efficacy of the administration of a single dose of polyclonal T_reg_ cells in controlling CIA in DBA/1 mouse model.^24^ Moreover, Sun et al have also demonstrated the efficacy of antigen specific T_reg_ cells in suppressing inflammation of CIA in C57BL/6 mouse model.^25^ However, these studies are limited by the durability of anti-inflammatory responses of antigen specific T_reg_ cells and the possibility of reversion to pathogenic effector T-cell type. Moreover, the studies did not show the efficacy of therapeutic adoptive T_reg_ cell therapy in controlling CIA in mice. Although previous studies have reported the efficacy of ACT of T_reg_ cells in autoimmune diseases^26^ our study is the first report showing the enhanced efficacy of combinatorial adoptive immunotherapy in C57BL/6 CIA mouse model. We have also shown that ACT of T_reg_ cells not only prevents CIA in mice, but also controls the progression of the disease when administered subsequent to the onset of the disease. More importantly, our study also provides an overview of the immune landscape in the CIA mouse model and shows how the adoption of our combinatorial strategy breaks immunological tolerance and ameliorates CIA.

Third, we demonstrate how the genetic tractability of BmΔ*vjbR* can be exploited to engineer vaccines with novel properties. Although in this work, we focused on metabolic reprogramming of the bacterium, we envision that future iterations of the BmΔ*vjbR::tnaA* vaccine will include engineered auto antigens (Ags) to boost persistent auto Ag-specific T_reg_ cell responses. Fourth, this work showed that a single administration of indole did not succeed as a stand-alone agent. However, this limitation was circumvented by indole delivery using our engineered bacterial vector. BmΔ*vjbR::tnaA* may offer improved pharmacodynamics for indole by sustained production levels and effects *in vivo* versus indole alone. Moreover, we showed that the separate individual beneficial effects of Bm*ΔvjbR* and indole act synergistically *in vivo*.

Finally, the natural localization of Bm*ΔvjbR* to leukocytes enabled BmΔ*vjbR::tnaA* to provide improved targeted indole delivery and a higher local effective indole concentration in contrast to the untargeted and transient nature of indole as a single agent. In sum, our work presents an attractive new avenue for the development of anti-autoimmunity/inflammation vaccines.

## Methods

### CIA induction

CIA was induced as described previously with minor modifications.^27^ Briefly, male C57BL/6 mice were injected with an emulsion of 100 µl of chick type II collagen (Chondrex; 100 µg) in Complete Freund’s Adjuvant (CFA; Chondrex) using a glass tuberculin syringe with 26-gauge needle. The mice were then assessed for development of joint inflammation and clinical arthritis score until Day 60 as described previously.^28^

### Indole treatment in CIA mouse

CIA was induced in male C57BL/6 mice. On Day 7 after the CIA induction, mice were intraperitoneally (*i.p*). injected with 20 mg/kg indole or corn oil (vehicle control).

### Bacterial culture

Bm∆*vjbR* or BmΔ*vjbR::tnaA* were cultivated and prepared for experimentation as previously described.^15^

### Engineering indole-producingBmΔ*vjbR::tnaA* strain

To generate an indole producing attenuated Bm∆*vjbR* strain, we cloned an *Escherichia coli* (*E. coli*) *tnaA* gene into a broad range bacteria expression plasmid (pBBR1MCS6Y)^29^ and transferred the plasmid to Bm∆*vjbR*.

### Indole Detection and quantification

The indole production by Bm∆*vjbR::tnaA* was detected by liquid chromatography–mass spectrometry (LC-MS). After 24 hr cultivation of the bacteria in Tryptic Soy Broth (TSB) medium at 37°C, the metabolites were extracted using ice-cold methanol for LC-MS assay. Liquid chromatography tandem mass spectrometry analysis was performed on a TSQ Altis triple quadrupole mass spectrometer (Thermo Scientific, Waltham, MA) coupled to a binary pump HPLC (Vanquish, Thermo Scientific). The indole concentration was measured using an indole assay kit following the manufacturer’s protocol (Sigma-Aldrich).

### BmΔ*vjbR*::tnaA treatment and ACT of T_reg_ cells

CIA was induced in male C57BL/6 mice. On Day 7 after the CIA induction, mice were intravenously (*i.v*). injected with 5.0 × 10^7^ live BmΔ*vjbR::tnaA* or PBS control. In the BmΔ*vjbR::tnaA* + T_reg_ cell combinatorial treatment group, mice (N = 5) were adoptively transferred with 2.5 × 10^6^ CD4^+^CD25^+^ T_reg_ cells derived from donor lymph nodes (LNs) and spleen of naive C57BL/6 mice, one week after the BmΔ*vjbR::tnaA* administration.

For treatment of established CIA, mice were injected with 5.0 × 10^7^ live BmΔ*vjbR::tnaA* or indole/PBS control on Day 21 post injection of CFA+Collagen followed by T_reg_ cell injection on Day 25.

### Enumeration of BmΔ*vjbR::tnaA* recovered from CIA mice

BmΔ*vjbR::tnaA* (5.0 × 10^7^) were *i.v*. injected into C57BL/6 mice and the bacterial distribution and survival were analyzed by colony forming unit (CFU) assay. The mice were sacrificed at 1, 3, 7, 14, and 21 dpi of bacteria. The spleen, liver, LNs and joints were homogenized and plated on Tryptic Soy Agar (TSA) plates supplemented with chloramphenicol. The CFU was enumerated after 3-day post-cultivation of the bacteria.

### Serum ELISA for detection of BmΔ specific IgG antibody

The CIA induced C57BL/6 mice were sacrificed at 1, 3, 7, 14, and 21 dpi of BmΔ*vjbR* and/or BmΔ*vjbR::tnaA* bacteria. Blood samples were collected from the mice and serum was isolated by coagulation of the blood at room temperature followed by centrifugation at 2,000 × g for 20 minutes. The serum sample was assayed for anti-BmΔ*vjbR* IgG antibody by using mouse *Brucella* antibody IgG ELISA kit following manufacturer’s instructions (AFG Scientific).

### Cytokine responses

BMDMs were seeded in 24-well plates at a concentration of 2.0 × 10^5^ cells/well in DMEM without antibiotics. After overnight culture, the cells were inoculated with Bm∆*vjbR* or Bm∆*vjbR::tnaA* bacteria at a multiplicity of infection of 20. At 24 h post-treatment, cellular supernatant was collected and analyzed for the presence of cytokines/chemokines by using a Proteome Profiler Mouse Cytokine Array Kit (R&D Systems, Inc.).

### Flow cytometric analysis

Cell staining and flow cytometric analysis were performed as described previously^15^ using the described labeling reagents. Briefly, surface and intracellular staining was performed on the single-cell suspensions and analyzed using LSR Fortessa cell analyzer (BD). The spleen, joints and LNs were also processed and stained similarly with antibodies listed in Table S2, and data was acquired on CyTEK aurora flow cytometer (Cytek Biosciences). For multiparametric analysis, the data were analyzed with FlowJo v10 and represented as heatmaps and tSNE plots.

### Histology and immunofluorescence

Mice were humanely sacrificed on day 60 after induction of CIA, and tissue sections were analyzed as previously described.^27^ Briefly, the hind foot paws and knees were removed and fixed in 10% formalin and decalcified in Formical-4 (Decal chemical, Tallman, NY). The fixed tissue sections were then stained with H&E and/or Safranin O fast green (Saf-O) stain. The H&E and Saf-O stained sections were then assessed by a semiquantitative system of 0 to 4 as described previously. Immunofluorescent staining and microscopy were performed on the deparaffinized sections by using FITC anti-mouse FoxP3 antibody (Ab) for T_reg_ cells and DAPI as nuclear stain.

### Statistical analysis

One-Way ANOVA, Student’s *t*-test, or Tukey’s multiple comparisons test was performed for statistical analysis between the groups. All analyses were performed in GraphPad Prism v9. A *p* value of <0.05 was considered statistically significant.

## Supplementary Material

Supplemental Material

## Key messages

What is already known about this subject?

- Bm∆*vjbR* has been shown to be safe in immunocompetent and immunocompromised animals.
- Microbiota-specific products and metabolites have novel immunologic properties.

What does this study add?

- Novel bacterial platform produce immunomodulatory metabolites that alter the immunological tolerance and the IME.

How might this impact on clinical practice or future developments?

- Combining a single dose of an engineered live attenuated bacterial therapeutic with ACT of T_regs_ can achieve potent therapeutic outcomes.

The use of engineered bacterial agents is an innovative and malleable approach to manage autoimmune and pro-inflammatory diseases.

## Data sharing statement

All data relevant to the study are included in the article.
