# Supplementary material for: A metabolically engineered bacterium controls autoimmunity and inflammation by remodeling the pro-inflammatory microenvironment
Source: Gut Microbes. 2022 Nov 20;14(1):2143222. doi: 10.1080/19490976.2022.2143222 (PMC9683044; doi:10.1080/19490976.2022.2143222)
Supplement: Supplemental Material [file KGMI_A_2143222_SM5853.pdf]

Fig. S1

a

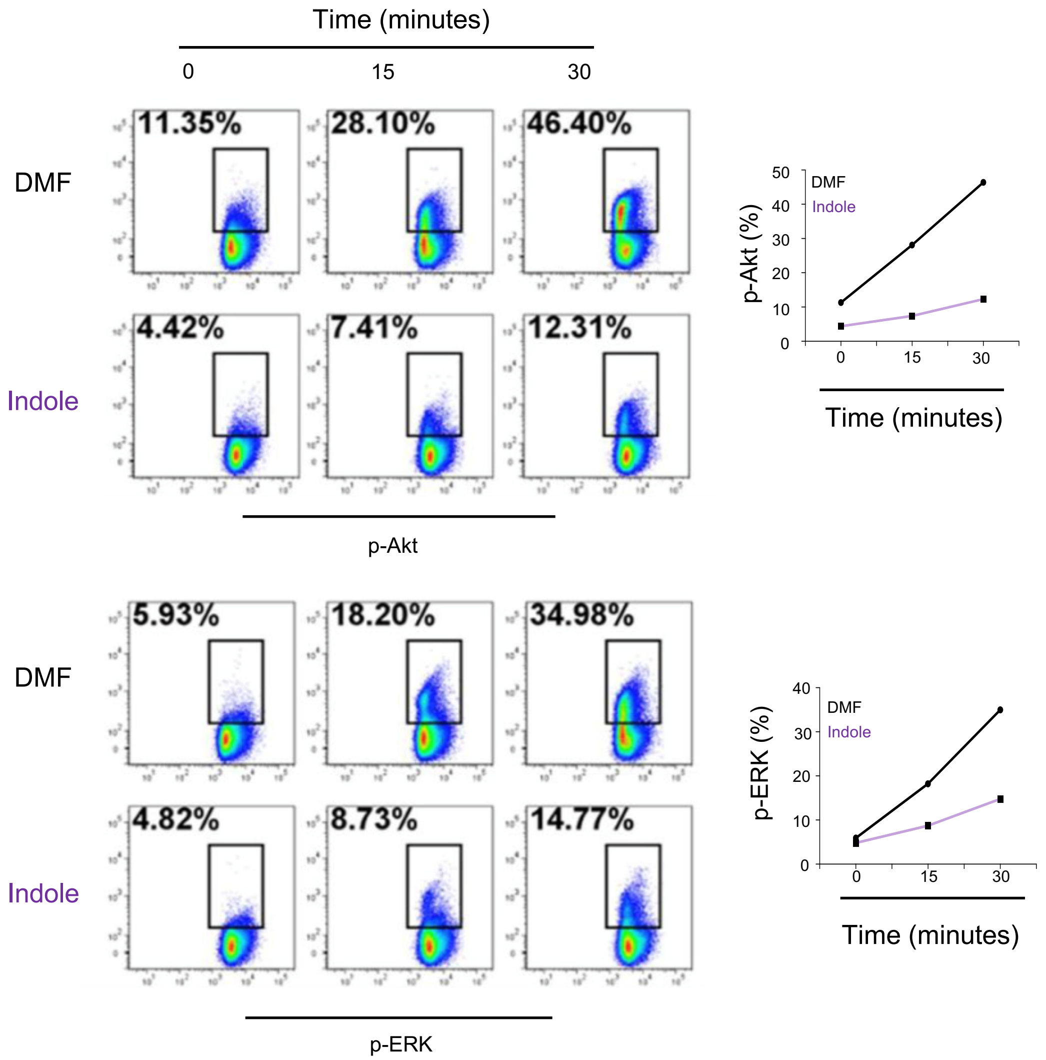
Time (minutes)
0
15
30
DMF
Indole
11.35%
28.10%
46.40%
4.42%
7.41%
12.31%
p-Akt
p-Akt (%)
DMF
Indole
Time (minutes)
5.93%
18.20%
34.98%
4.82%
8.73%
14.77%
p-ERK
p-ERK (%)
DMF
Indole
Time (minutes)

b

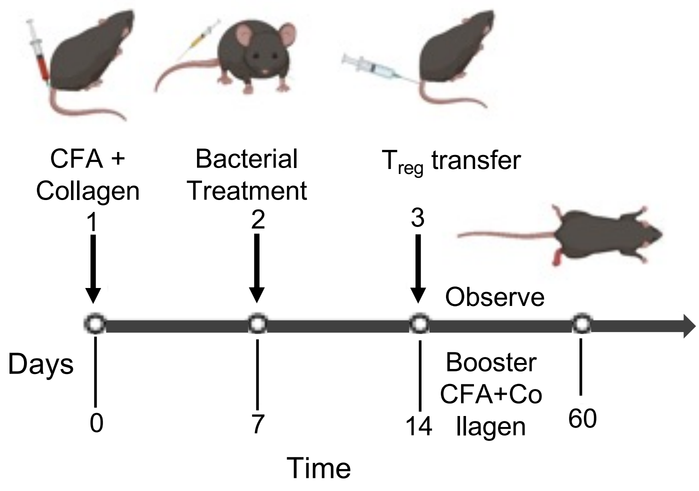
CFA +
Collagen
Bacterial
Treatment
T_{reg} transfer
1
2
3
Observe
Days
0
7
14
60
Booster
CFA+Co
llagen
Time

Fig S1: Indole suppresses activation of splenic CD11b$^+$ cells by suppressing Akt and ERK signaling pathway

a, Effect of indole (1mM) on the Akt and ERK signaling pathways in CD11b$^+$ cells (monocytes/macrophages) in different post incubation time. Figure depicts the flow cytometric dot-plot analysis and time-course graphical representation of phospho-Akt and phospho-ERK expression in CD11b$^+$ cells . **b,** Schematic diagram of murine CIA model used for results depicted in Fig 3c to Fig 3g. Mice were inoculated with an emulsion of CFA and collagen on Day 0 followed by bacterial treatment on Day 7 and Treg transfer on Day 14. A booster (repeat) injection of CFA + Collagen was administered to the mice on Day 21 to enhance CIA.

Fig. S2

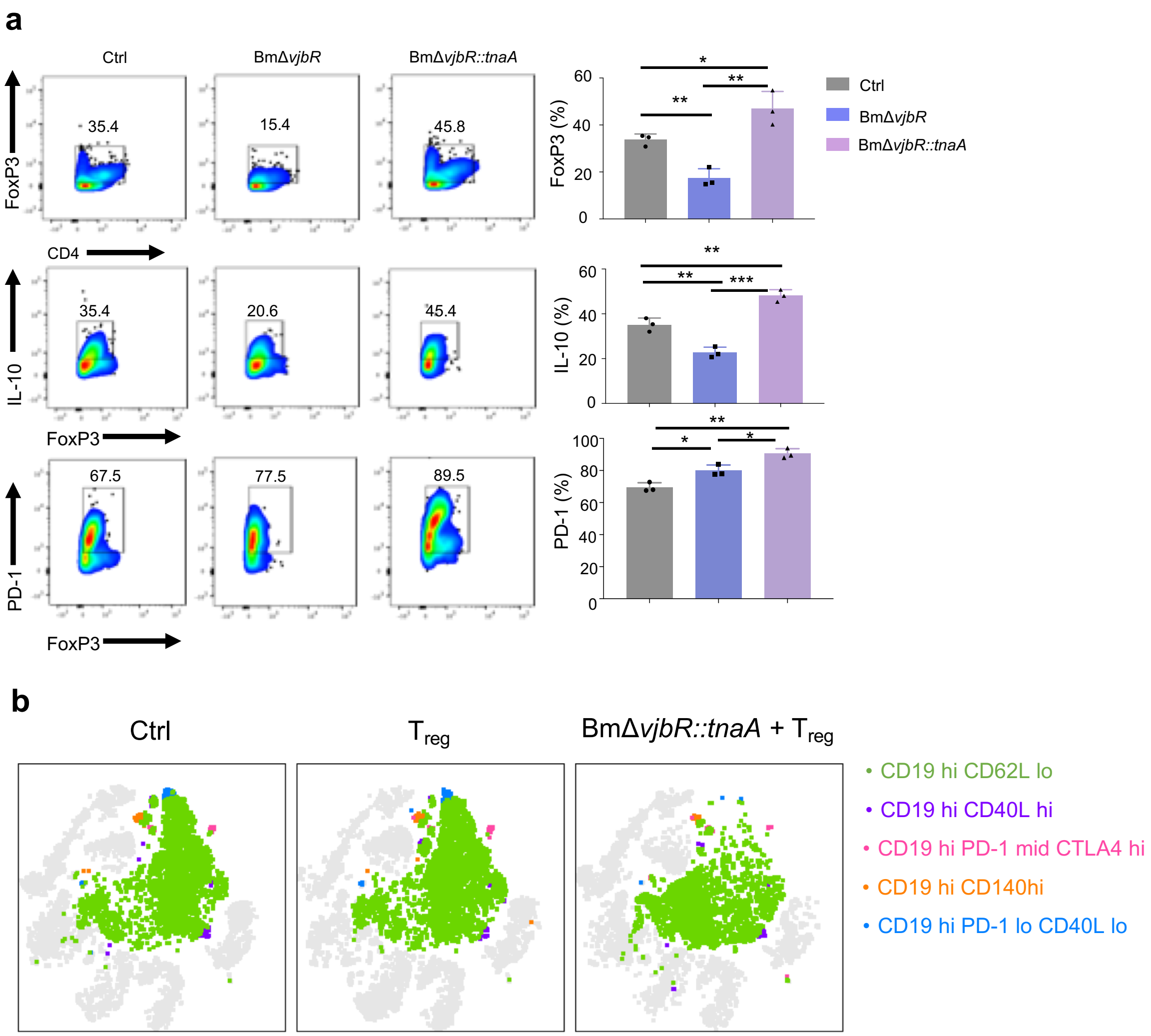

a
Ctrl
BmΔvjbR
BmΔvjbR::tnaA
FoxP3
CD4
35.4
15.4
45.8
IL-10
FoxP3
35.4
20.6
45.4
PD-1
FoxP3
67.5
77.5
89.5
FoxP3 (%)
IL-10 (%)
PD-1 (%)
Ctrl
BmΔvjbR
BmΔvjbR::tnaA
b
Ctrl
Treg
BmΔvjbR::tnaA + Treg
CD19 hi CD62L lo
CD19 hi CD40L hi
CD19 hi PD-1 mid CTLA4 hi
CD19 hi CD140hi
CD19 hi PD-1 lo CD40L lo

Fig. S2: BmΔ*vjbR::tnaA* activates T_{reg} cells and modulates B-cell mediated inflammation.

a, BMDMs infected with either BmΔ*vjbR::tnaA* or BmΔ*vjbR* were co-cultured with $CD4^+$ T cells from mouse LNs and spleen and activated by using anti-CD3/CD28 Abs. Flow cytometric dot-plot assay shows that BmΔ*vjbR::tnaA* treated BMDMs greatly promoted expression of FoxP3 and PD-1 and production of IL-10 in $CD4^+$ T cells. The dot-plots are followed by graphical representation from 3 independent experiments. **b,** The $CD19^+$ population is shown in the viSNE plots. Data represent means ± SD. Student's *t*-test or Tukey's multiple comparisons test was applied for statistical analysis. *, **, ***: significance at p<0.05, 0.01, 0.001.

Fig. S3

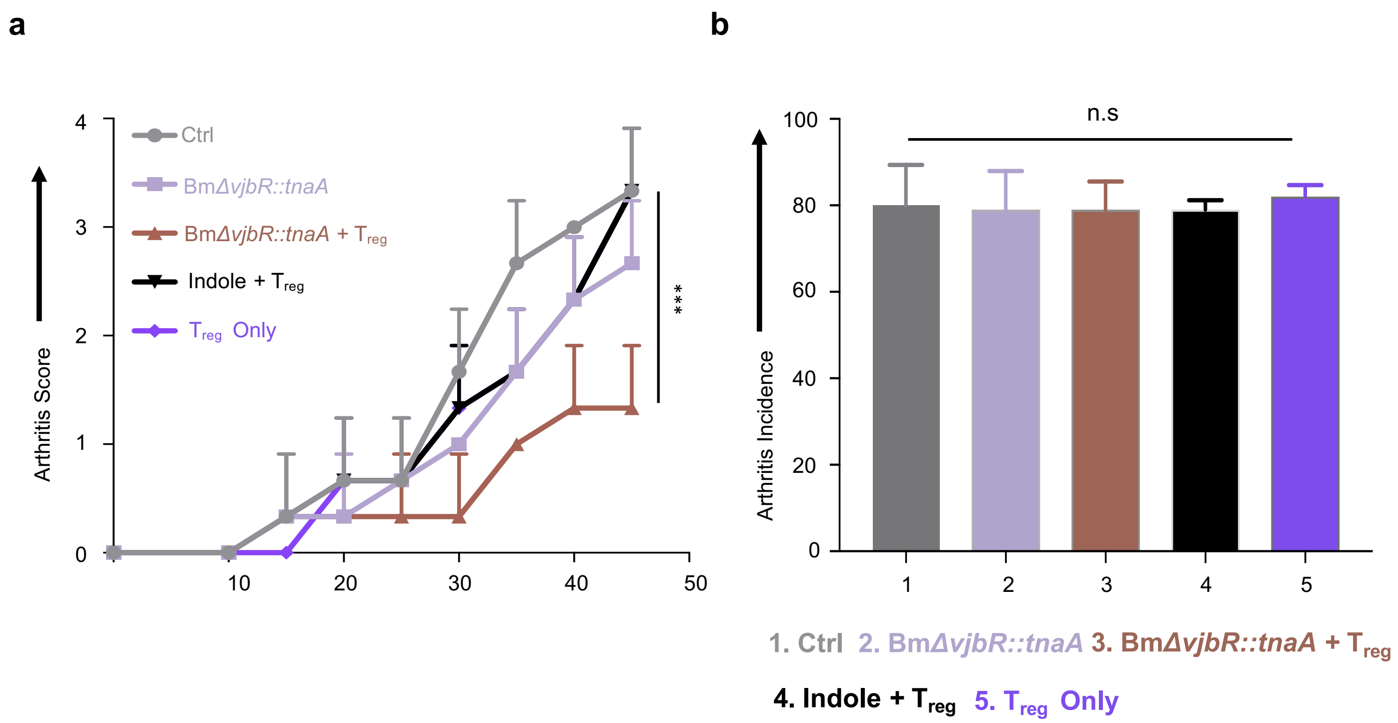

a
Arthritis Score
0
1
2
3
4
10
20
30
40
50
Ctrl
BmΔvjbR::tnaA
BmΔvjbR::tnaA + Treg
Indole + Treg
Treg Only
***
b
Arthritis Incidence
0
20
40
60
80
100
n.s
1
2
3
4
5
1. Ctrl 2. BmΔvjbR::tnaA 3. BmΔvjbR::tnaA + Treg
4. Indole + Treg 5. Treg Only

Fig. S3: BmΔ*vjbR::tnaA* in combination with T_{regs} delays the progression of CIA.

a, Arthritis Score and **b,** arthritis incidence derived from treatment of CIA mice with indicated treatment strategies. Mice were inoculated with an emulsion of CFA and collagen on Day 0 followed by BmΔ*vjbR::tnaA* (5×10^7 cfu) and/or indole treatment (20 mg/kg) on Day 21 followed by T_{reg} cell transfer on Day 25. The T_{reg} only group of mice received only T_{reg} cells without any other treatment. A booster (repeat) injection of CFA + Collagen was also administered to the mice on Day 21 to enhance CIA. Data represent means ± SD. Student's *t*-test or Tukey's multiple comparisons test was applied for statistical analysis. ***: significance at $p < 0.001$.

Table S1

Population	Cell type	Ctrl	T_{reg}	Bm*ΔvjbR ::tnaA* + T_{reg}
1	CD40L hi CD25 lo	8.6 %	18.7 %	72.7 %
2	CD25 hi Tim3 hi Ki67 hi	34.9 %	20.4 %	44.7 %
3	D45 lo CD44 mid	34.7 %	22.2 %	43.1 %
4	CD45 lo CD44 lo	34.3 %	27.9 %	37.8 %
5	CD44 hi CD160 mid	28.0 %	36.0 %	36.0 %
6	CD3 hi CD8hi CD25 lo	31.8 %	33.3 %	34.9 %
7	CD19 hi CD62L lo	32.7 %	33.6 %	33.7 %
8	CD4 hi CD25 lo	31.9 %	36.8 %	31.3 %
9	CD4 hi CD25 mid CTLA4 lo	37.0 %	32.0 %	31.0 %
10	CD25 hi PD-L1 hi	47.1 %	22.9 %	30.0 %
11	CD8 hi CD62L lo	38.2 %	32.4 %	29.4 %
12	CD19 hi CD40L hi	40.6 %	36.7 %	22.7 %
13	CD19 hi PD-1 mid CTLA4 hi	46.6 %	32.3 %	21.1 %
14	CD19 hi CD40L hi	32.1 %	47.4 %	20.5 %
15	CD25 hi CD140 hi	20.1 %	60.3 %	19.6 %
16	CCR7 hi PD-1 lo	53.1 %	28.0 %	18.9 %
17	CD4 mid PD-L1 hi	55.2 %	29.3 %	15.5 %
18	CD19 hi PD-1 lo CD40L lo	76.0 %	22.3 %	1.68 %

Supplementary Table 1. A list of different immune cell types which were differentiated using the tSNE analysis and their proportion in different treatment groups.

Table S2

Ab	Clone	Fluorophore	Source
**Ghost Dye Red 710**		Ghost Dye Red 710	Tonbo Biosciences
**CD45**	30-F11	FITC	Tonbo Biosciences
**CD8a**	53-6.7	BUV395	BD Biosciences
**CD4**	GK1.5	eFluor 450	Thermo Fisher Scientific
**CD3**	17A2	APC-Fire 810	BioLegend
**CD19**	1D3	BUV805	BD Biosciences
**MHC II**	M5/114.15.2	Violet Fluor 500	Tonbo Biosciences
**CD160**	7H1	APC	BioLegend
**PD-L1**	MIH5	Super Bright 780	Thermo Fisher Scientific
**CTLA-4**	UC10-4F10-11	PE-Cy7	Tonbo Biosciences
**PD-1**	29F.1A12	PE-Dazzle 594	BioLegend
**Lag-3**	C9B7W	BUV661	BD Biosciences
**TIM-3**	5D12/TIM-3	Brilliant Violet 605	BD Biosciences
**CD44**	IM7	PerCP	BioLegend
**CD62L**	HRL1	BUV737	BD Biosciences
**Ki-67**	B56	Brilliant Violet 711	BD Biosciences
**CCR7**	4B12	PE	BioLegend
**CD69**	H1.2F3	BUV563	BD Biosciences
**CD25**	PC61	Brilliant Violet 650	BioLegend
**CD40L**	MR1	BB700	BD Biosciences
**CD28**	37.51	BUV496	BD Biosciences

Supplementary Table 2: List of Abs used in CyTEK assay
